# Different Effects of Biologics on Systemic Bone Loss Protection in Rheumatoid Arthritis: An Interim Analysis of a Three-Year Longitudinal Cohort Study

**DOI:** 10.3389/fimmu.2021.783030

**Published:** 2021-12-20

**Authors:** Ming-Han Chen, Shan-Fu Yu, Jia-Feng Chen, Wei-Sheng Chen, The-Ling Liou, Chung-Tei Chou, Chung-Yuan Hsu, Han-Ming Lai, Ying-Chou Chen, Chang-Youh Tsai, Tien-Tsai Cheng

**Affiliations:** ^1^ Division of Allergy- Immunology- Rheumatology, Department of Medicine, Taipei Veterans General Hospital, Taipei, Taiwan; ^2^ Faculty of Medicine, National Yang Ming Chiao Tung University, Taipei, Taiwan; ^3^ Division of Rheumatology, Allergy, and Immunology, Department of Internal Medicine, Kaohsiung Chang Gung Memorial Hospital, Kaohsiung, Taiwan; ^4^ School of Medicine, College of Medicine, Chang Gung University, Taoyuan, Taiwan

**Keywords:** rheumatoid arthritis, osteoporosis, bone mineral density, tumor necrosis factor-α inhibitors, abatacept

## Abstract

**Objective:**

To compare changes in bone mineral density (BMD) in rheumatoid arthritis (RA) patients receiving three-year conventional synthetic disease-modifying anti-rheumatic drugs (csDMARD), tumor necrosis factor-α inhibitors (TNFi), and abatacept.

**Methods:**

Patients with RA were recruited from September 2014 to February 2021. Dual-energy X-ray absorptiometry was used to measure BMD at the femoral neck (FN), total hip (TH), and lumbar spine (L1-4) at enrollment and three years later. Changes in the BMD of each regimen group were analyzed. Multiple ordinary least squares regression was used with the dependent variables to develop a model to predict the change in BMD.

**Results:**

A total of 752 participants were enrolled and 485 completed the three-year follow-up period. Of these, 375 (Group I), 84 (Group II), and 26 (Group III) participants received csDMARDs, TNFi, and abatacept therapy, respectively. Considering both type of therapy and completion of the follow-up period, participants were divided into groups A (csDMARDs, n = 104), B (TNFi, n = 52), and C (abatacept, n = 26). Compared to baseline, BMD decreased significantly at FN (p = 0.003) and L1-4 (p = 0.002) in Group A and at L1-4 (p = 0.005) in Group B, but remained stable at all sites in Group C. In terms of regression-adjusted percent change in BMD, there was a significant difference seen at all measured sites between group C compared to both groups A and B (+0.8%, -2.7%, -1.8% at FN; +0.5%, -1.1%, -1.0% at TH; +0.8%, -2.0%, -3.5% at L1-4, respectively; all p < 0.05). Anti-osteoporosis therapy had a BMD-preserving effect in RA.

**Conclusion:**

Compared with csDMARDs and TNFi, abatacept may have a better BMD-preserving effect in RA. Anti-osteoporosis therapy can prevent systemic bone loss irrespective of RA therapy.

## Introduction

Rheumatoid arthritis (RA) is one of the most common forms of chronic inflammatory arthritis. This symmetrical polyarthritis mainly affects middle-aged females and leads to progressive joint destruction and loss of function (1). Osteoporosis is characterized by low bone mass, leading to bone fragility as well as a consequent increase in fracture risk (2). It is well known that patients with RA have an increased risk of developing osteoporosis. It has been reported that annual bone loss is greater in patients with active RA than in healthy patients (3). Compared with the general population, a two-fold increase in the frequency of osteoporosis in the spine was observed in RA patients (4). In addition, a meta-analysis revealed that the relative risk for bone fracture was higher among patients with RA than among those without RA (risk ratio 2.25) (5).

In recent years, a greater understanding of immunopathology has facilitated the development of biologic disease-modifying antirheumatic drugs (bDMARDs), which target specific components of the immune response and improve the clinical outcomes of RA (1). Inhibition of pro-inflammatory cytokines, such as tumor necrosis factor-α inhibitor (TNFi), seems to be effective in reducing disease activity and inhibiting bone loss in patients with RA (6–9). CTLA-4 Ig (Abatacept) is a fusion protein that regulates the T-cell co-stimulatory signal and is effective in attenuating disease activity and reducing joint damage in RA patients who have an inadequate response to methotrexate (10, 11). Previous research has demonstrated that abatacept can increase BMD at the femoral neck (FN) and is superior to that of other biologics in patients with RA (12).

In our previous investigation, we demonstrated that three-year biological/targeted synthetic DMARD (b/tsDMARD) treatment can prevent bone loss in RA patients and conventional synthetic DMARD (csDMARD) does not (13). However, the long-term effect in preserving BMD in patients with RA treated with TNFi or abatacept is unknown. The primary aim was to explore the BMD changes in patients with RA treated with csDMARD, TNFi, and abatacept *via* a three-year, real-world, observational, cohort study. The secondary aim was to investigate the synergistic effect of anti-osteoporosis therapy (AOT) on BMD in patients with RA receiving different therapies.

## Methods

### Study Population

This was a multi-center, three-year, real-world, observational cohort study. This study was approved by the Institutional Ethics Committee of Chang Gung Memorial Hospital, Kaohsiung (CGMHK) (approval number: 104-3530B, 106–0047 C) and Taipei Veterans General Hospital (TVGH) (approval number: 2018-04-006BC, 2020-09-013CC) and was conducted in accordance with the guidelines of the Declaration of Helsinki. Informed consent was obtained from all participants. The enrollment criteria included patients with RA who fulfilled the 1987 American College of Rheumatology (ACR) revised criteria (14) or the 2010 ACR/European League Against Rheumatism (EULAR) classification criteria (15), visited the rheumatology clinic at these two medical centers since September 2014, and received csDMARD, TNFi, or abatacept following the National Institute for Health and Care Excellence guidelines during the three-year observation period.

### Bone Mineral Density

The BMD at the FN, hip (total) (TH), and lumbar vertebra 1–4 (L1–4) of each participant were measured using dual-energy X-ray absorptiometry scanners (CGMHK, Delphi A; Hologic Corp., Waltham, MA, USA; TVGH, QDR 4500A; Hologic Inc., Waltham, MA, USA) upon enrollment and three years later.

Clinical and laboratory assays of the RA patients were recorded upon enrollment, including age, sex, body height, body weight, body mass index (BMI), and the presence of rheumatoid factor (RF) and anti-cyclic citrullinated peptide antibodies (ACPA). RA disease activity was measured using C-reactive protein (CRP), erythrocyte sedimentation rate (ESR), and Disease Activity Score in 28 joints based on ESR (DAS28-ESR) (16). Information on current medications at the time of enrollment was collected. In addition, risk factors for fragility fractures based on the FRAX tool were recorded. Considering all of these, the 10-year probabilities of major and hip fractures were calculated and recorded. Prescription of oral systemic glucocorticoids was recorded at baseline and during the study period, and was converted to a prednisolone equivalent dose. Baseline exposure was defined as current glucocorticoid usage of > 3 months before enrollment, noting and calculating the mean daily dose within the last three months. The mean daily dose of glucocorticoids during the observation period was determined by this equation: cumulative dose of glucocorticoid prescribed ÷ cumulative dispensing days during the three-year observation period.

### Statistical Analysis

Independent Student’s t-test was used to compare numerical data that exhibited normal distribution and Mann–Whitney U test was used for data that showed otherwise. Categorical variables were evaluated with chi-square test or Fisher’s exact test. Change in BMD of each participant from baseline was calculated using paired *t-test*. A one-way ANOVA or Kruskal-Wallis test was used to determine the significant difference between the three treatment groups in terms of parametric data. Multiple ordinary least squares (OLS) regression was used to assess the independent effects of drug treatment on the three dependent variables, controlling for age, gender, BMI (> or ≦ 24), disease duration, ACPA positivity, and baseline DAS28-ESR (> or ≦ 3.2), based on which the predicted value of the changes in BMD was calculated. Data are presented as mean ± SD or median (interquartile range, IQR) for normal and non-normal distribution datasets. The p*-*value was two-tailed and interpreted as significant when the value was < 0.05. Statistical analyses were performed using SPSS version 22.0 (IBM SPSS Statistics for Windows, IBM, Armonk, New York, USA).

## Results

### Patients

**Figure 1:**
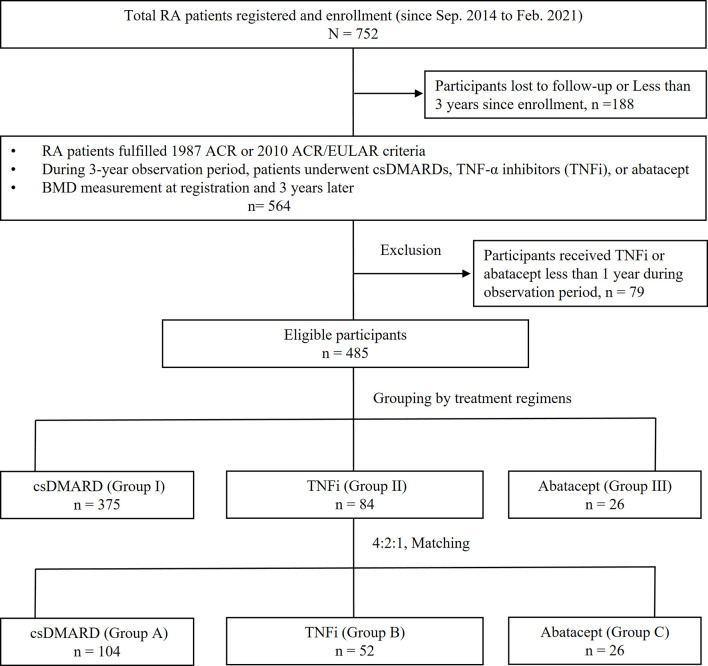
Disposition of participants and grouping. RA, rheumatoid arthritis; ACR, American College of Rheumatology; EULAR, European League Against Rheumatism collaborative initiative; conventional synthetic DMARD (csDMARD), including methotrexate, hydroxychloroquine, sulfasalazine, leflunomide, cyclosporine; TNFi, including etanercept, adalimumab, golimumab; BMD, bone mineral density.

A total of 752 participants were registered from September 2014 to February 2021, but only 485 participants completed the three-year follow-up period by the end of February 2021. The disposition of the participants is illustrated in **Figure 1**; 188 patients lost to follow-up or followed less than 3 years since enrollment, while 79 patients received TNFi or abatacept less than 1 year or switched to an another biologic agent with a different mechanism of action during observation period.

The demographics and clinical characteristics of the enrolled patients are shown in **Table 1**. Eligible participants were grouped into group I (n = 375, csDMARD), II (n = 84, TNFi, including etanercept, adalimumab, and golimumab), and III (n = 26, abatacept) or by regimens used during the observation period. Mean age and sex were not significantly different between the groups. The characteristics of RA disease entities were not obviously different among groups except for baseline disease activity (DAS28-ESR) (p < 0.001), three-year mean DAS28-ESR (p = 0.014), rate of positive RF (p = 0.001), rate of positive ACPA (p = 0.002), baseline glucocorticoid exposure (p < 0.001), and rate of cumulative exposure to glucocorticoids (p = 0.001). In addition, baseline BMD and risk factors for fragility fractures in the FRAX tool were comparable among the groups.

**Table 1:** Demographics and clinical characteristics of participants.

Group	All	I	II	III	P ^f^
	N = 485	n = 375	n = 84	n = 26	
Age (years)	57.6 ± 10.8	57.9 ± 10.7	57.3 ± 10.2	55.3 ± 12.8	0.490
Female, n (%)	420 (86.6)	325 (86.7)	72 (85.7)	23 (88.5)	0.957
Body weight (kg)	58.3 ± 11.1	58.0 ± 11.1	60.2 ± 11.8	56.7 ± 8.5	0.204
Body height (cm)	156.6 ± 7.2	156.3 ± 7.4	157.4 ± 5.9	158.2 ± 6.9	0.240
BMI (kg/cm2)	23.7 ± 3.9	23.7 ± 3.9	24.2 ± 4.3	22.8 ± 2.9	0.226
Factors associated with RA					
Disease duration (years),	12 (12)	12 (11.5)	13 (14)	7.5 (10.8)	0.086
Baseline DAS28-ESR	3.4 ± 1.3	3.2 ± 1.2	4.1 ± 1.4	4.9 ± 1.6	<0.001^*^
3-year mean DAS 28-ESR	3.1 ± 0.9	3.0 ± 0.9	3.3 ± 1.0	3.2 ± 1.0	0.014^*^
Rheumatoid factors, + (%)	324 (66.8)	236 (62.9)	67 (79.8)	21 (80.8)	0.001^*^
ACPA, + (%)	328/478 (68.6)	243/373 (65.1)	63/79 (79.7)	22/26 (84.6)	0.002^*^
FRAX risk factors ^a^					
Previous fracture +, n (%)	154 (31.8)	121 (32.3)	26 (31.0)	7 (26.9)	0.577
2^nd^ Osteoporosis +, n (%)	18 (3.7)	16 (4.3)	2 (2.4)	0 (0.0)	0.188
Glucocorticoid ^b^					
Baseline exposure +, n (%)	410 (84.5)	333 (88.8)	60 (71.4)	16 (61.5)	<0.001^*^
Baseline dose (mg/day),	5 (0.0)	5 (0.0)	5 (0.0)	5 (1.9)	0.504
Cumulative exposure + ^c^, n (%)	403 (83.1)	325 (86.7)	62 (73.8)	16 (61.5)	0.001^*^
Mean dose (mg/day) ^d^	5 (0.0)	5 (0.0)	5 (1.4)	5 (1.9)	0.786
Parent fractured hip +, n (%)	35 (7.2)	27 (7.3)	6 (7.1)	2 (7.7)	0.984
Osteoporosis ^e^, n (%)	106/460 (23.0)	84/369 (22.8)	13/68 (19.1)	9/23 (39.1)	0.165
Baseline BMD (g/cm^2^)					
Femoral neck	0.627 ± 0.111	0.628 ± 0.109	0.631 ± 0.120	0.598 ± 0.107	0.402
Total hip	0.785 ± 0.133	0.786 ± 0.128	0.789 ± 0.156	0.744 ± 0.123	0.309
lumbar spine (L1 – L4)	0.862 ± 0.156	0.855 ± 0.155	0.895 ± 0.163	0.856 ± 0.134	0.109
Current smoking +, n (%)	33 (6.8)	23 (6.1)	8 (9.5)	2 (7.7)	0.373
Alcohol +, n (%)	6 (1.2)	5 (1.3)	1 (1.2)	0	0.614
AOT +, n (%)	163 (33.6)	121 (32.3)	32 (38.1)	10 (38.5)	0.277
bisphosphonate	140 (28.9)	106 (28.3)	25 (29.8)	9 (34.6)	0.772
denosumab	26 (5.4)	21 (5.6)	4 (4.8)	1 (3.8)	0.896
SERM	6 (1.2)	2 (0.5)	4 (4.8)	0 (0.0)	0.006^*^
teriparatide	2 (0.4)	0 (0.0)	1 (1.2)	1 (3.8)	0.006^*^

2nd, secondary; ACPA, anti-citrullinated protein antibody; AOT, anti-osteoporosis therapy; BMD, bone mineral density; BMI, body mass index; csDMARD, conventional synthetic Disease-modifying antirheumatic drugs; DAS28-ESR, the disease activity score-28 for rheumatoid arthritis based on erythrocyte sedimentation rate; FRAX, fracture risk assessment tool; RA, rheumatoid arthritis; SERM, selective estrogen receptor modulator; TNFi, TNF-α inhibitors.

Data are presented as mean ± standard deviation, or median (interquartile range).

^a^defined as in FRAX tool.

^b^dose of glucocorticoid was converted to a prednisolone equivalent dose.

^c^defined as number and proportion (%) of participants who had ever received glucocorticoid therapy during the 3-year observation period.

^d^only for participants receiving glucocorticoid during observation period.

^e^defined as a T-score equal to −2.5 or less at femoral neck.

^f^comparison among group I, II, and III. *p < 0.05.

Matching the rate of glucocorticoid use across groups I to III to a ratio of 4:2:1, the groups were subdivided into groups A (n = 104, csDMARD), B (n = 52, TNFi), and C (n = 26, abatacept), respectively. The demographic and clinical characteristics of the participants are shown in **Table 2**. Mean age and sex were not significantly different between the groups. Body mass index was significantly different (p = 0.023). The characteristics of RA disease entities were not obviously different among groups except for baseline disease activity (DAS28-ESR) (p < 0.001), three-year mean DAS28-ESR (p = 0.004), and rate of positive ACPA (p = 0.013). The baseline BMD and risk factors for fragility fracture in the FRAX tool were comparable among groups after matching. Fifty-seven patients received AOT; 44, 5, 4, and 1 patient treated with bisphosphonate, denosumab, selective estrogen receptor modulators (SERM), and teriparatide alone, while one patient in group B received denosumab and teriparatide, another one patient in group B received bisphosphonate and SERM, and one patient in group C received bisphosphonate and denosumab during observation period. There was no significant difference in the percentage of patients on bisphosphonate, denosumab, and teriparatide between different groups, while more patients in group B received SERM when compared to those in other groups.

**Table 2:** Demographics and clinical characteristics of participants, after matching.

Group	All	A	B	C	P ^f^
	N = 182	n = 104	n = 52	n = 26	
Age (years)	57.5 ± 10.7	57.7 ± 10.5	58.1 ± 10.2	55.3 ± 12.8	0.531
Female, n (%)	159 (87.4)	93 (89.4)	43 (82.7)	23 (88.5)	0.500
Body weight (kg)	58.2 ± 10.6	56.8 ± 9.6	61.8 ± 12.6	56.7 ± 8.5	0.016^*^
Body height (cm)	157.4 ± 6.5	156.8 ± 6.8	158.1 ± 5.6	158.2 ± 6.9	0.425
BMI (kg/cm^2^)	23.5 ± 3.7	23.0 ± 3.3	24.7 ± 4.6	22.8 ± 2.9	0.023^*^
Factors associated with RA					
Disease duration (years)	10 (11.5)	10 (9)	11 (14.7)	7.5 (10.8)	0.170
Baseline DAS28-ESR	3.7 ± 1.5	3.1 ± 1.1	4.2 ± 1.5	4.9 ± 1.6	<0.001^*^
3-year mean DAS 28-ESR	3.0 ± 0.9	2.9 ± 0.8	3.4 ± 1.0	3.2 ± 1.0	0.004^*^
Rheumatoid factors, + (%)	142 (78.0)	78 (75.0)	43 (82.7)	21 (80.8)	0.508
ACPA, + (%)	133/179 (74.3)	69/104 (66.3)	42/49 (85.7)	22/26 (84.6)	0.013^*^
FRAX risk factors^a^					
Previous fracture +, n (%)	52 (28.6)	26 (25.0)	19 (36.5)	7 (26.9)	0.326
2^nd^ Osteoporosis +, n (%)	5 (2.7)	4 (3.8)	1 (1.9)	0 (0.0)	0.365
Glucocorticoid ^b^					
Baseline exposure +, n (%)	112 (61.5)	64 (61.5)	32 (61.5)	16 (61.5)	1.000
Baseline dose (mg/day),	5 (0.0)	5 (2.5)	5 (0.0)	5 (1.9)	0.186
Cumulative exposure + ^c^, n (%)	110 (60.4)	62 (59.6)	32 (61.5)	16 (61.5)	0.966
Mean dose (mg/day) ^d^,	5 (0.0)	5 (2.5)	5 (0.0)	5 (1.9)	0.255
Parent fractured hip +, n (%)	10 (5.5)	4 (3.8)	4 (7.7)	2 (7.7)	0.534
Osteoporosis ^e^, n (%)	40/166 (24.1)	23/102 (22.5)	8/41 (19.5)	9/23 (39.1)	0.204
Baseline BMD (g/cm^2^)					
Femoral neck	0.630 ± 0.116	0.632 ± 0.113	0.644 ± 0.129	0.598 ± 0.107	0.292
Total hip	0.787 ± 0.132	0.790 ± 0.119	0.802 ± 0.162	0.744 ± 0.123	0.211
lumbar spine (L1 – L4)	0.871 ± 0.156	0.858 ± 0.149	0.906 ± 0.175	0.856 ± 0.134	0.175
Current smoking +, n (%)	17 (9.3)	9 (8.7)	6 (11.5)	2 (7.7)	0.808
Alcohol +, n (%)	1 (0.5)	0 (0.0)	1 (1.9)	0 (0.0)	0.284
AOT +, n (%)	57 (31.3)	26 (25.0)	21 (40.4)	10 (38.5)	0.105
bisphosphonate	46 (25.3)	22 (21.2)	15 (28.8)	9 (34.6)	0.288
denosumab	7 (3.8)	3 (2.9)	3 (5.8)	1 (3.8)	0.677
SERM	5 (2.7)	1 (1.0)	4 (7.7)	0 (0.0)	0.034^*^
teriparatide	2 (1.1)	0 (0.0)	1 (1.9)	1 (3.8)	0.193

2^nd^, secondary; ACPA, anti-citrullinated protein antibody; AOT, anti-osteoporosis therapy; BMD, bone mineral density; BMI, body mass index; csDMARD, conventional synthetic Disease-modifying antirheumatic drugs; DAS28-ESR, the disease activity score-28 for rheumatoid arthritis based on erythrocyte sedimentation rate; FRAX, fracture risk assessment tool; RA, rheumatoid arthritis; SERM, selective estrogen receptor modulator; TNFi, TNF-α inhibitors.

Data are presented as mean ± standard deviation, or median (interquartile range).

^a^defined as in FRAX tool.

^b^dose of glucocorticoid was converted to a prednisolone equivalent dose.

^c^defined as number and proportion (%) of participants who had ever received glucocorticoid therapy during the 3-year observation period.

^d^mean daily dose of glucocorticoid only for participants receiving glucocorticoid during observation period.

^e^defined as a T-score equal to −2.5 or less at femoral neck.

^f^comparison among group A, B, and C. *p < 0.05.

### Comparison of BMD Changes With Baseline After Matching

**Figure 2:**
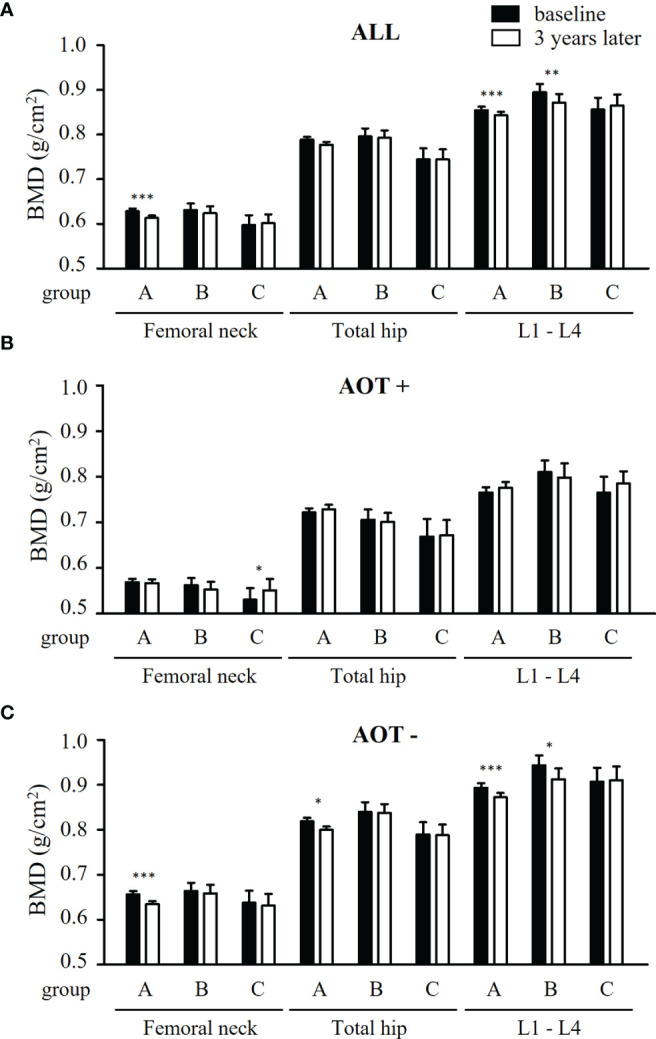
Comparison of BMD at baseline and 3 years later in all patients and patients with and without anti-osteoporosis therapy, after matching. **(A)** Differences of bone mineral density (BMD) at femoral neck, total hip, and lumbar spine 1-4 (L1-4) between baseline (black bars) and 3 years later (white bars) a in patients receiving conventional synthetic disease-modifying antirheumatic drugs (group A), TNF-α inhibitors (group B), or abatacept (group C). **(B, C)** Differences of BMD at three measured sites between baseline and 3 years later in group A - C, combined with anti-osteoporosis therapy (AOT) **(B)** or not **(C)**. *p < 0.05; **p < 0.01; ***p < 0.005.

Comparing baseline values of all participants, BMD at FN and L1-4 significantly decreased in group A (p *=* 0.003 and 0.002, respectively) (**Table 3** and **Figure 2A**). Although BMD of L1-4 significantly decreased in group B (p = 0.005), there were no significant changes seen at the three measured sites in group C. Changes in BMD in participants who received AOT are shown in **Table 3** and **Figures 2B, C**. BMD at FN, TH, and L1-4 in participants who received AOT remained stable in the three groups. An exemption to this was BMD at FN, which significantly increased compared to baseline in group C (p = 0.012). Participants in group A and without AOT showed significant declines in BMD at FN, TH, and L1-4 (p = 0.003, 0.027, and < 0.001, respectively) (**Figure 2C**). BMD of group B participants without AOT revealed a significant decline at–L1-4 (p = 0.010). BMD of group C participants without AOT remained stable at FN, TH, and L1-4 (p = 0.530, p = 0.888, and p = 0.741, respectively (**Figure 2C**).

**Table 3:** Comparison of BMD between baseline and 3 years later in each treatment group, after matching.

	Group								
	All			AOT +			AOT -		
	A^a^	B^a^	C^a^	A	B	C	A	B	C
n	101	41	24	24	14	9	77	27	15
BMD (g/cm^2^)									
FN									
base	0.632 ± 0.113	0.644 ± 0.129	0.598 ± 0.107	0.557 ± 0.072	0.560 ± 0.071	0.531 ± 0.076	0.655 ± 0.113	0.687 ± 0.132	0.638 ± 0.104
3-y	0.615 ± 0.108	0.633 ± 0.144	0.602 ± 0.098	0.551 ± 0.082	0.537 ± 0.090	0.551 ± 0.075	0.635 ± 0.107	0.635 ± 0.107	0.632 ± 0.100
P^b^	0.003	0.231	0.556	0.536	0.102	0.012	0.003	0.715	0.530
TH									
base	0.791 ± 0.119	0.802 ± 0.162	0.744 ± 0.123	0.722 ± 0.110	0.723 ± 0.116	0.669 ± 0.116	0.812 ± 0.114	0.842 ± 0.170	0.790 ± 0.106
3-y	0.781 ± 0.112	0.797 ± 0.148	0.744 ± 0.110	0.736 ± 0.112	0.707 ± 0.113	0.672 ± 0.101	0.795 ± 0.109	0.844 ± 0.143	0.788 ± 0.092
p^b^	0.151	0.665	1.000	0.265	0.287	0.899	0.027	0.881	0.888
L1-4									
base	0.858 ± 0.149	0.906 ± 0.175	0.856 ± 0.134	0.758 ± 0.123	0.799 ± 0.120	0.766 ± 0.104	0.891 ± 0.142	0.977 ± 0.170	0.907 ± 0.124
3-y	0.839 ± 0.149	0.879 ± 0.191	0.865 ± 0.123	0.758 ± 0.127	0.775 ± 0.139	0.785 ± 0.080	0.866 ± 0.147	0.949 ± 0.192	0.911 ± 0.121
p^b^	0.002	0.005	0.277	0.989	0.168	0.164	<0.001	0.010	0.741

AOT+, received anti-osteoporosis therapy; AOT-, did not receive anti-osteoporosis therapy; base, baseline; BMD, bone mineral density; 3-y, 3 years later; FN, femoral neck; L1-4, lumbar vertebrae 1-4; TH, total hip.

Data are presented as mean ± standard deviation.

^a^A, csDMARD; B, TNFi; C, abatacept;

^b^BMD comparison between baseline and 3 years later.

### Differences in Percent Change of BMD Among Groups After Matching

**Figure 3:**
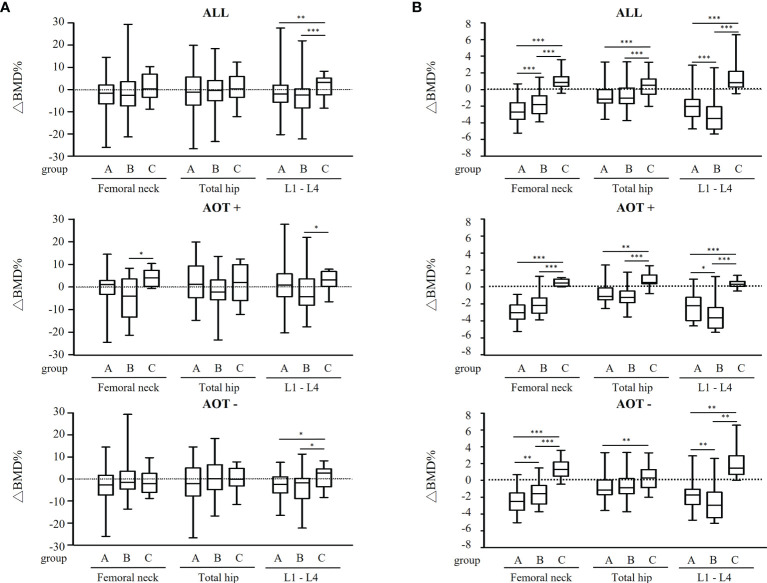
Regression-adjusted percentage change in BMD from baseline in each treatment group, after matching in all participants and participants with or without anti-osteoporosis therapy. Unadjusted **(A)** and regression-adjusted **(B)** percentage of change in BMD at femoral neck, total hip, and lumbar spine 1-4 (L1-4) after 3 years in patients receiving conventional synthetic disease-modifying antirheumatic drugs (group A), TNF-α inhibitors (group B), or abatacept (group C), combined with anti-osteoporosis therapy (AOT) or not. Box-and-whisker plots showed the median, interquartile range, and extreme values. *p < 0.05; **p < 0.01; ***p < 0.005.

After three years, percent changes in BMD (ΔBMD%) at FN and TH were not significantly different among groups except at L1-4 (p = 0.026) after matching in all participant groups (**Table 4** and **Figure 3A**). ΔBMD% at L1-4 in group C was significantly different from that in group A (median [interquartile range], +3.2 [7.6] % vs. -2.0 [7.7] %, p = 0.007) and group B (+3.2 [7.6] % vs. -2.5 [8.6] %, p = 0.002), respectively. ΔBMD% at FN, TH, and L1-4 were not obviously different among participants with AOT (p = 0.083, 0.356, 0.232, respectively) or without (p = 0.246, 0.478, 0.068, respectively) (**Table 4**). However, ΔBMD% at FN (+4.1 [7.0] % vs. -4.0 [16.9] %, p = 0.033) and L1-4 (+3.2 [6.6] % vs. -4.3 [11.7] %, p = 0.020) in group C were significantly different compared to group B participants with AOT. Meanwhile, ΔBMD% at L1-4 in group C was significantly different from group A (+2.8 [8.1] % vs. -2.4 [7.3] %, p = 0.033) or group B (+2.8 [8.1] % vs. -1.8 [9.1] %, p = 0.034) in participants without AOT.

**Table 4:** Regression-adjusted percentage change in BMD from baseline in each treatment group, after matching.

	Group	A^a^	B^a^	C^a^	P^c^
			N	△BMD %^b^	
Total	FN	101	41	24	
	unadjusted	-1.6 (8.5)	-2.5 (3.8)	0.3 (10.4)	0.146
	adjusted ^d^	-2.7 (2.0)	-1.8 (2.1)	0.8 (1.2)	<0.001
	TH	101	41	24	
	unadjusted	-1.2 (12.7)	-0.4 (9.1)	0.3 (9.4)	0.790
	adjusted	-1.1 (1.6)	-1.0 (1.9)	0.5 (1.8)	<0.001
	L1-4	99	50	25	
	unadjusted	-2.0 (7.7)	-2.5 (8.6)	3.2 (7.6)	0.026
	adjusted	-2.0 (2.0)	-3.5 (2.7)	0.8 (1.9)	<0.001
AOT +	FN	24	14	9	
	unadjusted	1.1 (6.1)	-4.0 (16.9)	4.1 (7.0)	0.083
	adjusted	-3.0 (1.7)	-2.2 (1.8)	0.5 (0.9)	<0.001
	TH	24	14	9	
	unadjusted	1.2 (14.0)	-2.2 (8.8)	1.9 (15.9)	0.356
	adjusted	-1.1 (1.4)	-1.2 (1.4)	0.5 (1.0)	0.005
	L1-4	25	20	1.9	
	unadjusted	0.8 (10.1)	-4.3 (11.7)	3.2 (6.6)	0.232
	adjusted	-2.2 (2.7)	-3.6 (2.4)	0.3 (0.6)	<0.001
AOT -	FN	77	27	15	
	unadjusted	-2.7 (8.9)	-1.5 (8.2)	-2.1 (8.6)	0.246
	adjusted	-2.7 (2.0)	-1.8 (2.1)	0.8 (1.2)	<0.001
	TH	77	27	15	
	unadjusted	-2.0 (12.7)	0.1 (11.3)	-0.1 (7.9)	0.478
	adjusted	-1.1 (1.6)	-1.0 (1.9)	0.5 (1.8)	0.014
	L1-4	74	30	16	
	unadjusted	-2.4 (7.3)	-1.8 (9.1)	2.8 (8.1)	0.068
	adjusted	-2.0 (2.0)	-3.5 (2.7)	0.8 (1.9)	<0.001

AOT+, received anti-osteoporosis therapy; AOT-, did not receive anti-osteoporosis therapy; FN, femoral neck; L1-4, lumbar vertebrae 1-4; TH, total hip BMD, bone mineral density; FN, femoral neck; L1-4, lumbar vertebra 1-4; TH, total hip.

Data are presented as median (interquartile range).

^a^A, csDMARD; B, TNFi; C, abatacept.

^b^△BMD%: [(BMD 3 years later – BMD at baseline)/BMD at baseline] × 100%.

^c^Comparison of △BMD% among groups at each site

^d^Predicted change in BMD was calculated by multiple regression analysis after adjusting age, gender, BMI (> or ≦ 24), disease duration, anti-cyclic citrullinated peptide antibody positivity, and baseline DAS28-ESR (> or ≦ 3.2).

Next, predicted change in BMD was calculated by the multiple regression analysis after adjusting age, gender, BMI, disease duration, ACPA positivity, and baseline DAS28-ESR (**Figure 3B** and **Table 4**). A decline in BMD in group A and B (-2.7 [2.0] % and -1.8 [2.1] % at FN, -1.1 [1.6] % and -1.0 [1.9] % at TH, -2.0 [2.0] % and -3.5 [2.7] % at L1-4, respectively), but BMD in group C remained (+0.8 [1.2] %, +0.5 [1.8] %, and +0.8 [1.9] % at FN, TH, and L1-4, respectively) when compared to 3 years earlier. Regardless of AOT, regression-adjusted ΔBMD% at all measured sites in group C was significantly different from that in group A and B (all p < 0.05), except regression-adjusted ΔBMD% at TH were not significantly different among group B and C participants without AOT (p = 0.088). Regression-adjusted ΔBMD% at FN and L1-4 in group B was significantly different from that in group A (both p < 0.05), while the regression-adjusted ΔBMD% at FN became less statistically different among participants with AOT (p = 0.053).

## Discussion

Current investigation demonstrated that AOT can prevent bone loss irrespective of regimen used, while in patients without AOT, participants who received csDMARD had the most obvious bone loss at all sites. Participants who received abatacept therapy demonstrated stable BMD at all sites, irrespective taking AOT therapy or not. This investigation revealed that abatacept may have a better systemic bone loss protection effect in RA patients than the other two regimens.

The b/tsDMARD not only demonstrated better control of disease activity, but also showed a better bony erosion protection effect than csDMARD in RA patients (17–26). Our previous investigation revealed that long-term b/tsDMARD therapy demonstrated a potentially beneficial effect on the protection of systemic bone loss (13). However, previous investigations regarding the effect of biologics on the prevention of systemic bone loss in RA have only focused on changes in bone turnover markers (18–21), over a short-term observation period (25, 26), and with a lack of an adequate control group (25, 27). In addition, the long-term effects of bDMARDs with different mechanisms of action on systemic bone loss in patients with RA remain obscure.

CTLA-4 is a negative regulator that inhibits antigen-specific immune responses after T-cell activation by interfering with the interaction of CD28 on T-cells and CD80/86 on antigen-presenting cells, including dendritic cells, macrophages, and B-cells (28, 29). Axmann et al. found that CTLA-4 directly binds to osteoclast precursor cells and dose-dependently inhibits receptor activator of nuclear factor-κB ligand (RANKL)-mediated osteoclastogenesis in an animal model (30). However, in human studies, abatacept demonstrated a controversial effect on the protective effect of bone loss in RA patients (12, 31).

Pro-inflammatory cytokines, such as TNF-α, IL-6, and IL-1, play a key role in the pathogenesis of RA and have been approved as treatment targets (7). The interaction between inflammation and osteoporosis has been previously described (32). In synovial fibroblasts, TNF-α upregulates Dickkopf-1 (DKK1), a negative regulator of Wnt signaling, leading to suppression of bone formation (8). In addition, TNF-α directly inhibits osteoblast differentiation and function (9). Therefore, the inhibition of pro-inflammatory cytokines seems to be an effective bone-protecting principle in patients with RA. Indeed, previous studies have demonstrated that TNFi is potentially beneficial for bone loss (17–26). However, the results of these studies were based on their influence on bone turnover markers (18–21). Furthermore, a significant increase in serum parathyroid hormone was found in RA patients undergoing TNFi treatment, which might promote bone resorption and blunt the anti-osteoporotic effect of TNFi (33). Taking together, whether or not there is a substantial improvement in BMD in RA patients treated with TNFi remains controversial (22–24).

As TNFi and abatacept were the first two regimens launched and are the most commonly prescribed biologics in Taiwan, we compared the protective effect to systemic bone loss of these two biologics with csDMARD. The current investigation revealed that abatacept therapy arrested systemic bone loss at FN, TH, and L1-4 in a three-year follow-up period. In addition, it seems that abatacept exhibited bone loss protective effect whether on AOT or not in RA patients. Furthermore, in terms of percent change of BMD, compared with TNFi, abatacept demonstrated a superior effect at FN and L1-4 and at L1-4 in patients who were taking AOT or not taking AOT, respectively.

It remains unclear why abatacept had a more favorable effect on BMD than csDMARD or TNFi. Our previous investigation demonstrated that adequate control of disease activity can protect bone loss in RA patients (34). Meanwhile, it has been demonstrated that positive ACPA status is associated with a differential treatment response to abatacept, but not TNFi (35). Furthermore, abatacept treatment showed differential efficacy in RA patients with higher ACPA titers (36). Previous studies reported that RA patients positive for ACPA had a lower BMD and a higher 10-year probability of fracture as evaluated by FRAX^®^ (37, 38). Patients with RA receiving abatacept therapy demonstrated a decline in ACPA titers or ACPA seronegative conversion effects (39). In addition, compared with group I or II, the rate of ACPA positivity was significantly higher in the abatacept group (group III) (p = 0.002) in our cohort. Based on the aforementioned findings, we hypothesize that adequate control of disease activity (DAS28-ESR) between baseline and during the observation period (4.9 ± 1.6 and 3.2 ± 1.0, p < 0.001) in the abatacept group and higher rate of ACPA in group III could partly explain the discrepancy in the effect on BMD among the groups. It is also well known that low BMI is one of risk factors of systemic bone loss or osteoporosis (40). In current investigation, abatacept group, although had lower BMI, increased more BMD than other groups after 3 years. After adjusting age, gender, BMI, disease duration, ACPA positivity, and baseline DAS28-ESR, predicted change in BMD at all measured sites in group C remained better than in other groups, suggesting that the better effect of abatacept on prevention of systemic bone loss in RA when compared to other comparison regimens.

As the current study is a real-world investigation, we did not exclude participants who received AOT during the observation period to elucidate the interaction of DMARD therapy and AOT in terms of bone protective effects. Participants who did not receive AOT had significant bone loss at all sites in group A and at the lumbar vertebrae in group B. AOT had a protective effect against bone loss in all groups at all sites. These results suggest that AOT plays the most important role in bone loss protection in RA patients receiving either csDMARD or biologics.

A strength of the current investigation is that it is a longitudinal, real-world, observational, registry, cohort study. We measured and recorded the characteristics of the disease entity at baseline and serial disease activity, which could potentially influence BMD changes during the study period. In addition, most previous studies were single-arm studies without an adequate control group (25, 27). Our initial investigation revealed a significant difference in the rate of glucocorticoid use among groups. As glucocorticoid use is a well-known risk factor for bone loss, we performed a 4:2:1 matching for glucocorticoid use to exclude the confounding effect of glucocorticoids, which had not been done in previous investigations. Furthermore, to adjust confounders of osteoporosis, multiple regression analysis was used to develop a model to predict the change in BMD. Finally, this study is the first investigation to explore the long-term systemic bone loss protective effect among DMARDs with different mechanisms of action to elucidate the effects of biologics on RA patients.

The current study has some limitations. As it was a real-world study, we did not exclude participants who had already received biologics before enrollment. Hence, we could not exclude the residual effect of previous medications on BMD after enrollment. However, we excluded participants with biologic switching during the observation period to avoid additional confounding effects. In addition, we did not compare the bone loss protective effect between abatacept and biologics other than TNFi, eg. tocilizumab, rituximab. So far, we could not know which biologics is the best one to prevent bone loss in RA patients. Fracture prevention is a hard outcome to measure in osteoporosis studies, and we could not compare the effect of fracture prevention among the treatment groups owing to the short duration and relatively small sample size of our study.

## Conclusion

RA patients receiving long-term abatacept illustrated a better bone loss protective effect than patients receiving csDMARD or TNFi. Anti-osteoporosis therapy has a vital protective effect on bone loss irrespective of regimens for RA therapy used. Further studies are needed to clarify whether abatacept or other biologics could prevent fragility fractures in patients with RA.

## Data Availability Statement

The original contributions presented in the study are included in the article/supplementary material. Further inquiries can be directed to the corresponding author.

## Ethics Statement

The studies involving human participants were reviewed and approved by Chang Gung Memorial Hospital, Kaohsiung and Taipei Veterans General Hospital. The patients/participants provided their written informed consent to participate in this study.

## Author Contributions 

Study concept: M-HC and T-TC. Study design: M-HC, S-FY, and T-TC. Data analysis: M-HC, S-FY, T-TC. M-HC, S-FY, J-FC, W-SC, T-LL, C-TC, C-YH, H-ML, Y-CC, C-YT, and T-TC were responsible for interpretation of the data and for drafting and revising the manuscript. All authors contributed to the article and approved the submitted version.

## Funding

This work was supported by grants CMRPG8F1111 and CMRPG8K0441 from Chang Gung Memorial Hospital (https://www.cgmh.org.tw/), which sponsored the cost of data collection, gathering, processing, and publication. The funder had no role in the study design, data analysis, decision to publish, nor manuscript preparation.

## Conflict of Interest

The authors declare that the research was conducted in the absence of any commercial or financial relationships that could be construed as a potential conflict of interest.

## Publisher’s Note

All claims expressed in this article are solely those of the authors and do not necessarily represent those of their affiliated organizations, or those of the publisher, the editors and the reviewers. Any product that may be evaluated in this article, or claim that may be made by its manufacturer, is not guaranteed or endorsed by the publisher.

## Abbreviations

ACPA, anti-cyclic citrullinated peptide antibodies; ACR, American College of Rheumatolo*gy; AOT*, anti-osteoporosis therapy; bDMARD, biologic disease-modifying antirheumatic drug; BMI, body mass index; CRP, C-reactive protein; csDMARD, conventional synthetic DMARD; DAS28-ESR, Disease Activity Score in 28 joints based on erythrocyte sedimentation rate; DKK1, Dickkopf-1; EULAR, European League Against Rheumatism; FN, femoral neck; FRAX, fracture risk assessment tool; L1–4, lumbar vertebra; RA, rheumatoid arthritis; RANKL, receptor activator of nuclear factor-κB ligand; RF, rheumatoid factor; TH, total hip; TNFi, tumor necrosis factor-α inhibitor; tsDMARD, targeted synthetic DMARD.
